# Efficact of prion decontamination of medical instruments using alkaline and enzymatic detergents

**DOI:** 10.1186/1753-6561-5-S6-P314

**Published:** 2011-06-29

**Authors:** A Sava, S Kritzler

**Affiliations:** 1 Research, Novapharm Research, Sydney, Australia; 2 Research, Novapharm Research Australia, Sydney, Australia

## Introduction / objectives

The recent confirmation of Alzheimer’s disease transmissibility, together with the large amount of data regarding the infectious nature of prions and other protein misfolding diseases, highlights the need for reliable protein decontamination of all reusable medical instruments.

In order to decontaminate medical instruments of potentially infectious proteins to sterilisation assurance levels similar to current sterilisation techniques, the decontamination protocol should not only remove but, preferably, break up (hydrolyse) the infectious protein into small, water soluble fragments of less than 3-5 KDa.

Currently, the proteins are routinely removed during the instruments exposure to either alkaline or enzymatic detergents during washing.

## Methods

Using straightforward stoichiometric equations and available literature data combined with our own results on kinetics of enzymatic and alkaline protein hydrolysis we assessed the quantities of dissolved and surface proteins that could be hydrolized during a standard CSDD wash cycle.

## Results

We estimate that, during a common CSSD wash cycle, a formulated multi-enzymatic detergent with validated enzyme activity can cleave 200-1000 times greater number of peptide bonds than a comparable alkaline detergent.

We discuss numerous adjustments to the results that take into account protein folding, chemosorption on the wide spectrum of instrument surfaces, availability of cleaving sites, proteolysis resistance, etc.

## Conclusion

A reliable removal of potentially infectious proteins and peptides from medical instruments is technically achievable via a standard 10-min, 60C CSSD wash with enzymatic detergents containing validated protein cleaving properties. The alkaline detergents, at realistic use levels, cannot assuredly hydrolyse proteins.

## Disclosure of interest

None declared.

